# The effect of aqueous extract of *Prunus dulcis* on tibial bone healing in the rabbit

**DOI:** 10.1186/s13018-021-02498-z

**Published:** 2021-06-07

**Authors:** Nima Anaraki, Amir Hossein Beyraghi, Abbas Raisi, Farshid Davoodi, Ghasem Farjanikish, Amin Bigham Sadegh

**Affiliations:** 1 Department of Clinical Sciences, Faculty of Veterinary Medicine, Lorestan University, Khorramabad, Iran; 2 Department of Surgery and Diagnostic Imaging, Faculty of Veterinary Medicine, Urmia University, Urmia, Iran; 3 Department of Pathobiology, Faculty of Veterinary Medicine, Lorestan University, Khorramabad, Iran; 4 Department of Veterinary Surgery and Radiology, School of Veterinary Medicine, Shiraz University, Shiraz, Iran

**Keywords:** Bone regeneration, Fracture healing, Prunus dulcis, Almond, Bone healing, Tibial fracture

## Abstract

**Background:**

Bone fractures are medical emergencies that require prompt intervention to help return bone to its normal function. Various methods and treatments have been utilized to increase the speed and efficiency of bone repair. This study aimed to investigate the treatment effects of *Prunus dulcis* aqueous extract on tibial bone healing in rabbits.

**Methods:**

All animals were distributed in five groups with six rats in each group, including the sham group, the control group in which tibial lesion was made and received distilled water, treatment groups with 150 mg kg^−1^, 300 mg kg^−1^ doses of *Prunus dulcis* extract, and osteocare treated group. Biochemical blood factors including calcium, phosphorus, and alkaline phosphatase (on days 0, 10, 30, and 50), biomarkers of oxidative stress such as GPx, CAT, and MDA (on days 10 and 30), radiological evaluation, histopathological parameters, and osteocalcin immunohistochemical expression were assessed.

**Results:**

The data showed calcium levels in the treatment groups increased significantly from day 10 to day 50, respectively, and blood phosphorus levels decreased from day 10 to day 50 in the treatment groups. Alkaline phosphatase initially increased and then decreased in treatment groups. In the treatment groups, GPx and CAT levels significantly increased, and the serum amount of MDA reduced. The best antioxidant results were related to the extract-treated group with a higher dose. Radiographic score was significantly higher in the treatment groups than the control group on day 30. Based on the pathological findings, the healing occurred faster in the extract-treated group with a higher dose. Osteocalcin expression was significantly higher in the control group than that in the treatment groups.

**Conclusions:**

Treatment with *Prunus dulcis* extract with a dosage of 300 mg/kg accelerated tibial bone healing in rabbits.

**Graphical abstract:**

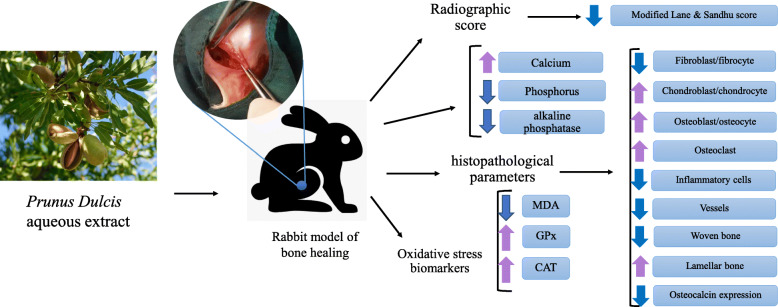

## Introduction

Bone is a tissue whose injuries have been reported frequently, and bone injuries can severely affect the quality of life [1]. Orthopedic surgeons constantly confront several challenges when curing the fractures, including malunion, nonunion, and delayed union bone fractures [2]. Good reduction and alignment lead to bone healing. However, in some cases, bone grafts are employed to help fast healing with better results [3]. There are various types of bone grafts, such as autografts, allografts, and xenografts. Autografts are defined as gold standards in literature. The most common complication of autografts is disorders of the donor site. Application of the allografts is also limited owing to host immune responses [4]. Bone healing possesses three phases, including the inflammatory stage, repair stage, and remodeling [5]. The bone healing process is regulated by plenty of agents such as vitamin D, calcitonin hormone, platelet-derived growth factors, fibroblast growth factor, and alkaline phosphate level [6].

The use of herbs to treat diseases in humans and animals is growing in many countries [7]. According to the World Health Organization (WHO), about 80% of people worldwide trust traditional medicine and the use of alternative and complementary medicine, including herbal extracts [8]. Almond, with the scientific name of *Prunus dulcis*, is a plant native to Iran and the Middle East. This tree is now grown in many countries with various climates [9]. *Prunus dulcis* is rich in lipids, proteins, fiber, and other minerals. Moreover, like other plant compounds, *Prunus dulcis* has several antioxidant compounds, such as benzoic acid and flavonoids [10]. Plenty of benefits for the body health were described owing to *Prunus dulcis* consumption in the previous studies such as balancing the level of serum lipid and glucose, protective effects in cardiovascular disorders, obesity, and diabetes [11]. Evaluating the mineral composition of *Prunus dulcis* revealed that calcium, phosphorus, potassium, and magnesium are the most important minerals existing in the extract [11].

The present study was aimed to examine the protective effects of *Prunus dulcis* aqueous extract in improving bone healing in a rabbit model.

## Material and methods

### Preparation of aqueous extract of *Prunus dulcis*

To prepare the aqueous extract of *Prunus dulcis* (almond), the *Prunus dulcis* were completely dried and powdered. Then, 100 grams of the prepared powder was added to 1 L of boiling water and heated for 10 min. The resulting solution was kept on a shaker for 48 h. The solution was then passed through a filter three times and put in an oven with 37 °C temperature to be concentrated. The resulting extract was transferred to a freezer with a temperature of – 20 °C and kept in the freezer until being used in experiments. Afterward, it was dissolved in sterile distilled water according to the desired dose. Prepared samples of *Prunus dulcis* extract were analyzed using the gas chromatography-mass spectrometry (GC-MS) method for determining the number of different substances available in the extract at the Central Laboratory of Lorestan University. The result of the GC-MS is represented in Table 1.

**Table 1:** Gas chromatography–mass spectrometry (GC-MS) of *Prunus Dulcis*

Compound	Percent
Benzene alcohol	38.82
Benzoic acid	9.98
Propanediol	9.54
Butanediol	6.73
Pentanol-3-methyl-2	5.36
Geranyl acetate	5.22
Phenyl ethyl alcohol	1.65
Hexadecanoic acid	1.11
Benzyl acetate	0.98
Terpinolene	0.89
Eicosane	0.87
Thymol	0.72
Carvacrol	0.57

### Animals and groups

In the present study, 30 albino New Zealand adult male rabbits with an average age of 7–8 months and a weight range of 2.5–3 kg were utilized. The animal ethics committee approved all the experiments within this study. All rabbits were housed in appropriate standard cages with a temperature of 26 ± 0.5 °C and humidity of 55 ± 0.4%. All animals were fed with commercial rabbit food and water ad libitum and received human care according to Institutional Animal Care guidelines. All rabbits were randomly divided into five groups, with six rats in each group as follows:

- Group 1: No surgical procedure was done, and rabbits received a daily oral gavage of the extract with a dosage of 200 mg/kg (sham group).
- Group 2: Tibial lesion was made, and daily oral gavage of distilled water was performed (control group).
- Group 3: Tibial lesion was made, and animals received a daily oral gavage of *Prunus dulcis* extract with a dosage of 150 mg/kg (PDE 150).
- Group 4: Tibial lesion was made, and animals received a daily oral gavage of *Prunus dulcis* extract with a dosage of 300 mg/kg (PDE 300).
- Group 5: Tibial lesion was induced surgically, and a daily oral gavage of Osteocare syrup (1 ml per day) (VITABIOTICS, London, UK) was performed (OC).

### Surgical procedure

Rabbits were anesthetized using a combination of ketamine hydrochloride (25 mg/kg, KETASET, Zoetis, NJ, USA) and xylazine (Dutch Farm International, The Netherlands). Rabbits received intravenous cefazoline (20 mg/kg, iv, marginal ear vein) as a prophylactic antibiotic 30 min before surgical procedures. After induction of anesthesia, the left hindlimb of the rabbits was shaved and disinfected using betadine scrub 7.5%. A 3-cm incision was made on the left hindlimb using a scalpel blade No. 10 to access the tibial bone. Regional muscles covering the tibia were precisely incised using scissors, and the tibia was exposed. A 5-ml hole was created on the tibial bone [12], and after cleaning the surgical site and washing the site with sterile normal saline, the muscles were sutured (VICRYL,1-0), and the skin was sutured with suture materials (NYLON, 1-0). After the operation, the rabbits in each group were returned to certain cages and kept separately. All animals received intramuscular cefazolin (20 mg/kg) for 3 days, and the surgical site was examined daily for swelling and infections. No evidence of surgical related complications and infections was observed a week following surgery. The rabbits were kept for 50 days following surgery and got daily oral gavage, based on the treatment groups. Eventually, the rabbits were euthanized using an overdosage of sodium thiopental (200 mg/kg), and bone samples were taken and were fixed in 10% formalin buffer solution. Samples fixed in formalin were sent to the pathology laboratory of Lorestan University for histopathological evaluation.

### Biochemical analysis of blood samples

Blood samples were taken on days 0 (before surgery), 10, 30, and 50 to examine the healing process among different experimental groups of the study. The samples were taken from the marginal ear vein of rabbits and were centrifuged at 5000 rpm for ten minutes to separate the serum. Afterward, serum samples were stored in a freezer at – 20 °C until being used in subsequent experiments. To measure calcium, phosphorus, and alkaline phosphatase, Biosystems kits (Barcelona, Spain) were employed according to the manufacturer’s protocols.

#### Blood calcium measurement

Serum calcium was measured using the Arsenazo III enzymatic method. In this method, the blood serum reacts with the Arsenazo enzyme and forms a color solution as a product. Blood calcium was then measured at 650 nm using a spectrophotometer.

#### Blood phosphorus measurement

The enzymatic method of Phosphomolybdate in an acidic medium was employed to measure blood phosphorus. The product of this reaction is molybdenum blue, which was detected in a spectrophotometer with a wavelength of 660 nm, and the amount of phosphorus was calculated according to the kit protocol.

#### Alkaline phosphatase measurement

Since the alkaline phosphatase enzyme does not have a specific substrate, various methods were employed in previous studies to measure this enzyme. Herein p-Nitrophenyl Phosphate was utilized as a substrate. p-Nitrophenyl Phosphate reacts with Diethylamine (DEA) and produces a colored agent named p-nitrophenol. p-nitrophenol is measured in the 409 nm using a spectrophotometer, which is directly related to alkaline phosphatase activity.

### Oxidative damage assessment

Glutathione peroxidase (GPx) and catalase (CAT) enzymes and the amount of malondialdehyde (MDA) in the serum samples were examined using Asan biochemical kits (Khorramabad, Iran). All methods were carried out based on the protocols described by the manufacturer.

### Radiographic assessment

Radiographs were taken on days 10, 30, and 50 to evaluate the tibial bone healing process. The modified Lane and Sandhu method was employed to score bone radiographs from 0 to 4 grades as follows: score 0, no sign of bone formation; score 1: bone formation with 25% defect filling; score 2: bone formation with 50% defect filling; score 3: bone formation with 75% defect filling; score 4: bone formation with 100% defect filling [13].

### Histopathological evaluation

For histopathological evaluation, following the samples’ fixation in 10% formalin buffer solution, they were placed in 15% nitric acid for 48 h to absorb calcium from the samples. Subsequently, tissue samples were placed in the Auto-technicon Tissue Processor machine (NY, USA), and dehydration and transparency of samples were automatically performed. Then, tissue samples were immersed in paraffin, and blocks were prepared. A rotary microtome was employed to make 4–5 μm sections from the tissue blocks. Provided sections with specified thickness were mounted on microscopic slides, and the hematoxylin and eosin (H&E) method was employed to stain the slides. Afterward, a skilled pathologist evaluated the slides using a light microscope with different magnifications. All slides were assessed and graded for histopathological parameters, including fibroblasts/fibrocytes, chondroblasts/chondrocytes, osteoblasts/osteocytes, osteoclast, inflammatory cells, vessels, woven bone, and lamellar bone.

### Immunohistochemical evaluations

Prepared tissue blocks were deparaffinized and hydrated. Then, immunohistochemistry staining protocol was performed based on previous studies [14]. In the present study, osteocalcin monoclonal antibody (Ab13420, Abcam, Cambridge, UK) was used to detect osteoblasts and bone formation in different experimental study groups.

### Statistical analysis

Statistical analysis of the data was performed using SPSS software (IBM SPSS Statistics version. 22). A one-way ANOVA test was used to compare biochemical parameters, oxidative stress biomarkers, and pathological indicators, and a p value less than 0.05 (*p* ≤ 0.05) was considered statistically significant.

## Result

### Blood biochemical parameters

**Fig. 1:**
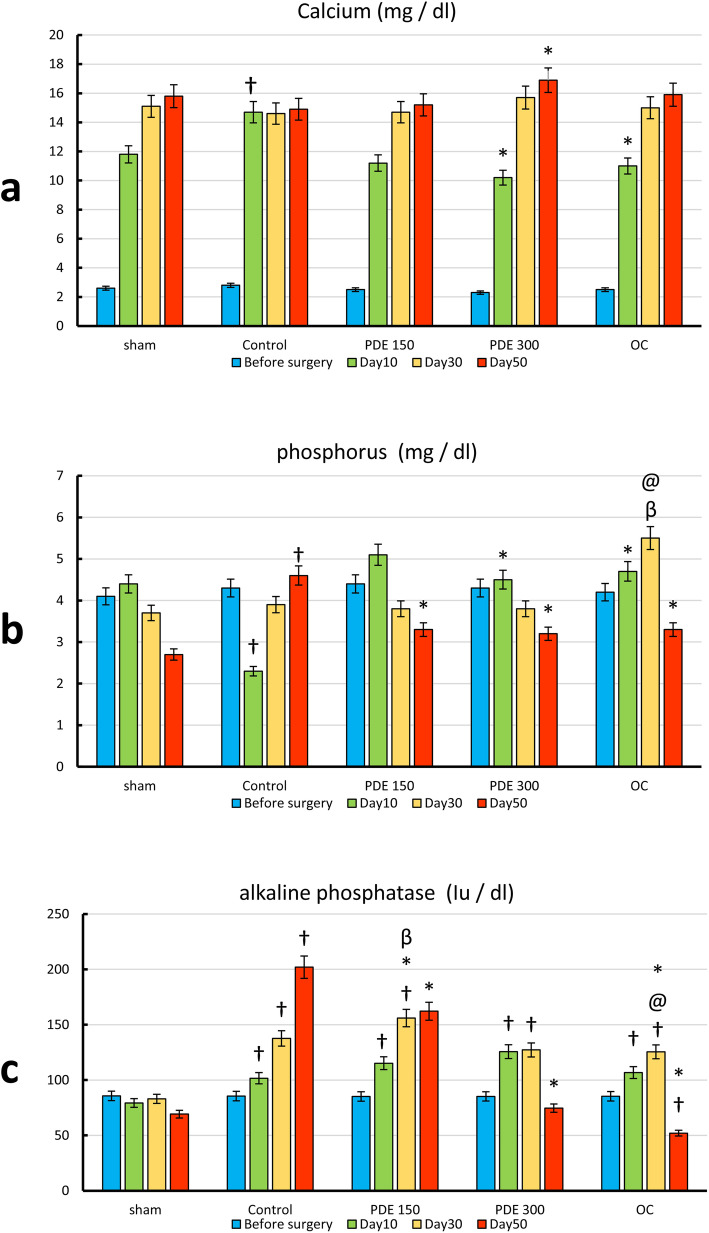
Levels of calcium, phosphorus, and alkaline phosphatase in experimental groups of the study on days 0, 10, 30, and 50. **a** Calcium level in the serum. **b** Phosphorus level in the serum. **c** The level of alkaline phosphatase. † Significant difference compared to sham group. * Significant difference compared to control group. @ Significant difference compared to group 150 mg. β Significant difference compared to 300 mg

Figure 1 represents the experimental data on calcium, phosphorus, and alkaline phosphatase. Figure 1a shows data regarding blood calcium levels in different study groups on days 0, 10, 30, and 50. According to results on day 10 in all groups except for the control group, the blood calcium level was reduced. In all treatment groups, blood calcium levels increased on day 30 and continued to increase till day 50. Based on the graphs, treatment with PDE 300 and osteocare significantly reduced calcium level on day 10 compared to the control group (*P* < 0.05). Treatment with *Prunus dulcis* extract with 300 mg/kg dosage significantly increased calcium level in comparison to the control group (*P* < 0.05).

The results obtained from the preliminary analysis of phosphorus are presented in Fig. 1b. There was a significant difference in serum phosphorus levels between the control group and the sham group on days 30 and 50 (*P* < 0.05). On day 10, a significant difference was observed in the amount of phosphorus in the PDE 300 and OC groups compared to the control group (*P* < 0.05). On day 30, treatment with osteocare remarkably increased the level of phosphorus compared to both groups treated with PD extract (*P* < 0.05), but no significant difference was observed between the treatment groups and the control group (*P* > 0.05). The amount of phosphorus notably decreased in all treatment groups compared to the control group on day 50 (*P* < 0.05). Overall, on day 10 in all groups, except for the control group there was an increment in the level of phosphorus, and on day 30, phosphorus level was reduced in all groups except for the control and the OC group.

As shown in Fig. 1c, levels of alkaline phosphatase in the control group were remarkably higher than that in the sham group on days 10, 30, and 50 (*P* < 0.05). On day 10, no significant difference was observed between the treatment groups and the control group (*P* > 0.05). On day 10, no significant difference was observed between the treatment groups and the control group (*P* > 0.05). On day 30, levels of alkaline phosphatase were significantly lower in the PDE 300 and OC groups in comparison to the PDE 150 group (*P* < 0.05). On day 50, in all treatment groups, level of the alkaline phosphatase was notably reduced compared to the control group (*P* < 0.05).

### Oxidative damage results

**Fig. 2:**
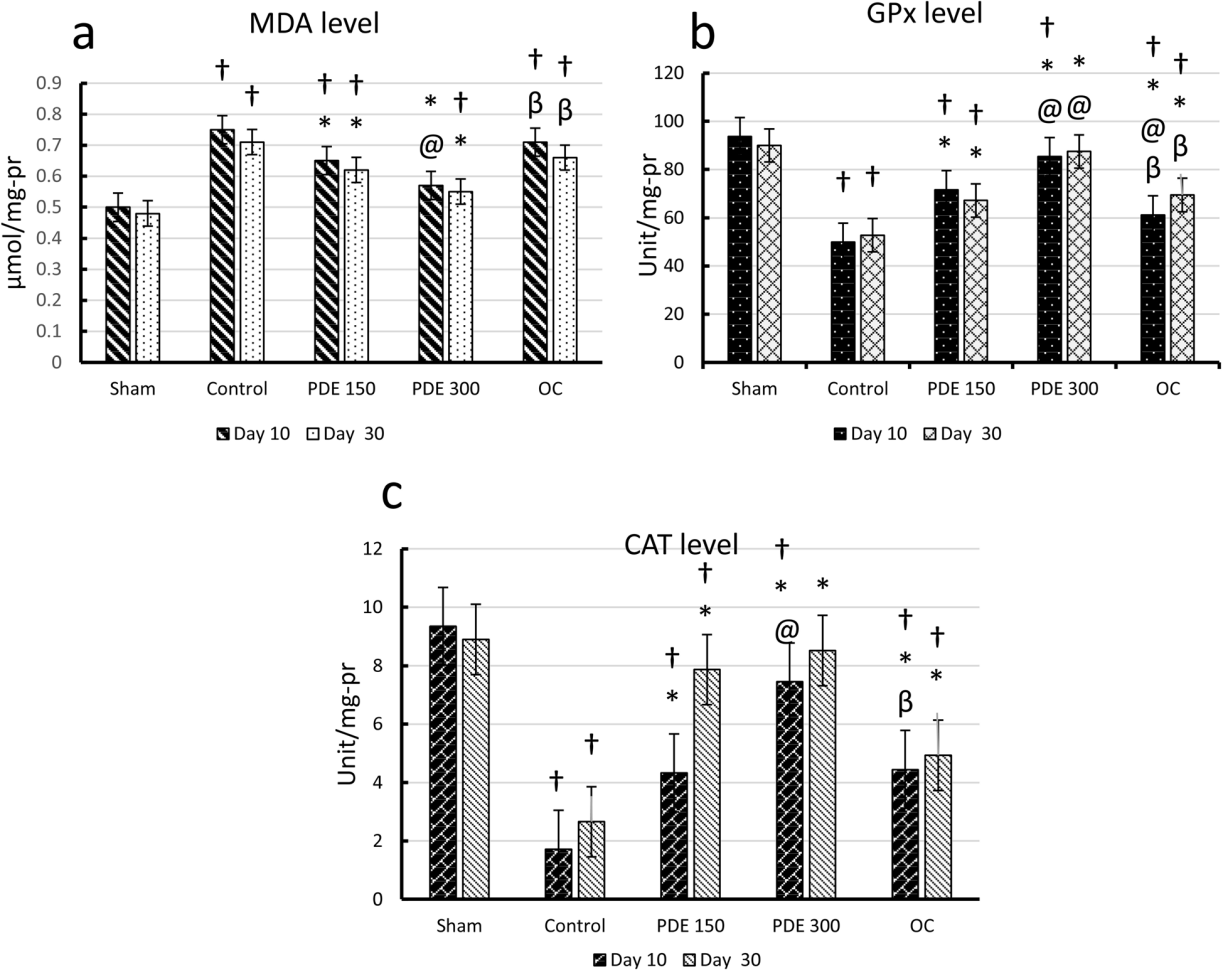
Oxidative stress biomarkers in different study groups on days 10 and 30. **a** MDA level in the serum. **b** GPx level in the serum. **c** CAT level in the serum. † Significant difference compared to sham group. *Significant difference compared to control group. @ Significant difference compared to group 150 mg. β Significant difference compared to 300 mg

Figure 2a presents the results obtained from the preliminary analysis of MDA. As can be seen from the graph, on days 10 and 30, there was a significant difference between extract-treated groups and the control group (*P* < 0.05). PDE 300 group remarkably reduced the MDA level compared to the PDE 150 group on day 10 (*P* < 0.05). Levels of MDA in the PDE 300 group were significantly lower than that in the OC group both on days 10 and 30 (*P* < 0.05).

Figure 2b shows GPx levels in different study groups on days 10 and 30. A significant difference was observed in all treatment groups compared to the control group on days 10 and 30 (*P* < 0.05). Treatment with PDE 300 notably increased the level of GPx compared to the PDE 150 and OC groups (*P* < 0.05). There was a significant difference between the OC group and the PDE 150 group on day 10 (*P* < 0.05).

Figure 2c indicates the catalase activity. All treatment groups had a significant difference with the control group on days 10 and 30 (*P* < 0.05). In the PDE 300 group level of CAT was higher than that in the PDE 150 and the OC groups on day 10 (*P* < 0.05).

### Radiographic findings

**Fig. 3:**
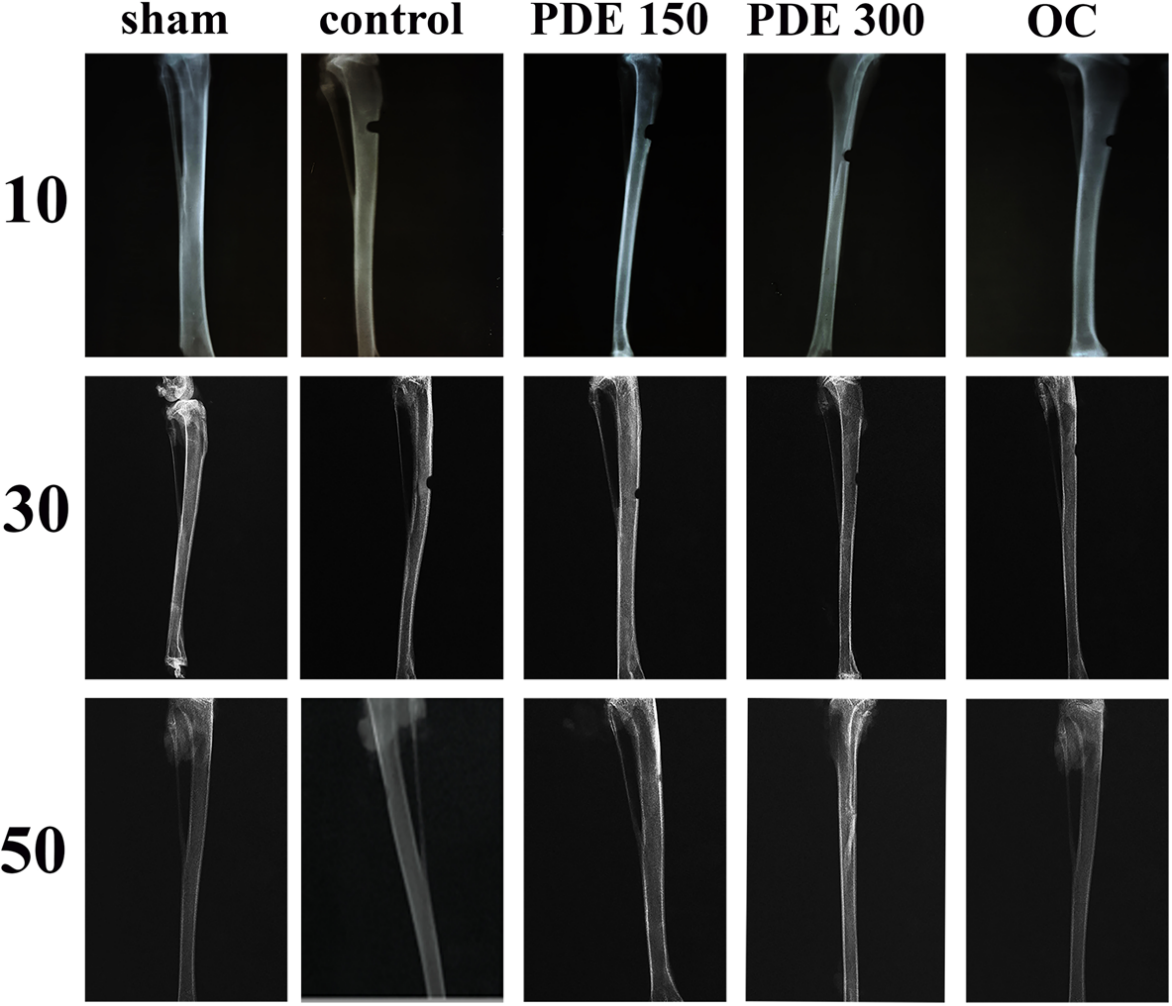
Radiographs of the tibial bone on days 10, 30, and 50 in different experimental study groups

Figure 3 represents radiographs of tibial bone on days 10, 30, and 50 in various groups. Table 2 indicates the radiographic scores of the tibial bone regeneration in different groups. As can be seen from the table, on day 10 no significant difference was observed between the treatment groups and the control group (*P* > 0.05). On day 30, a significant difference was observed between all treatment groups and the control group (*P* < 0.05). On day 50, the difference was not significant between the treatment groups and the control group (*P* > 0.05).

**Table 2:** Comparison of median of radiographic scores (modified Lane and Sandhu) in different study groups on days 10, 30, and 50

Groups	Days		
	10 MEDIAN(MIN-MAX)	30 MEDIAN(MIN-MAX)	50 MEDIAN(MIN-MAX)
Control	0(0-0) ^a^	1(0-1) ^b^	3(1-3) ^f^
Sham	4(4-4)	4(4-4)	4(4-4)
PDE 150	0(0-0.5) ^a^	2(1-2) ^c^	3(3-4) ^f^
PDE 300	0(0-1) ^a^	3(1-3) ^d^	4(3-4) ^f^
OC	0(0-1) ^a^	3(2-3) ^e^	4(3-4) ^f^

Data are represented as median (min-max). Different letters in each column indicate a significant difference with the control group (*P* < 0.05)

### Histopathological results

**Fig. 4:**
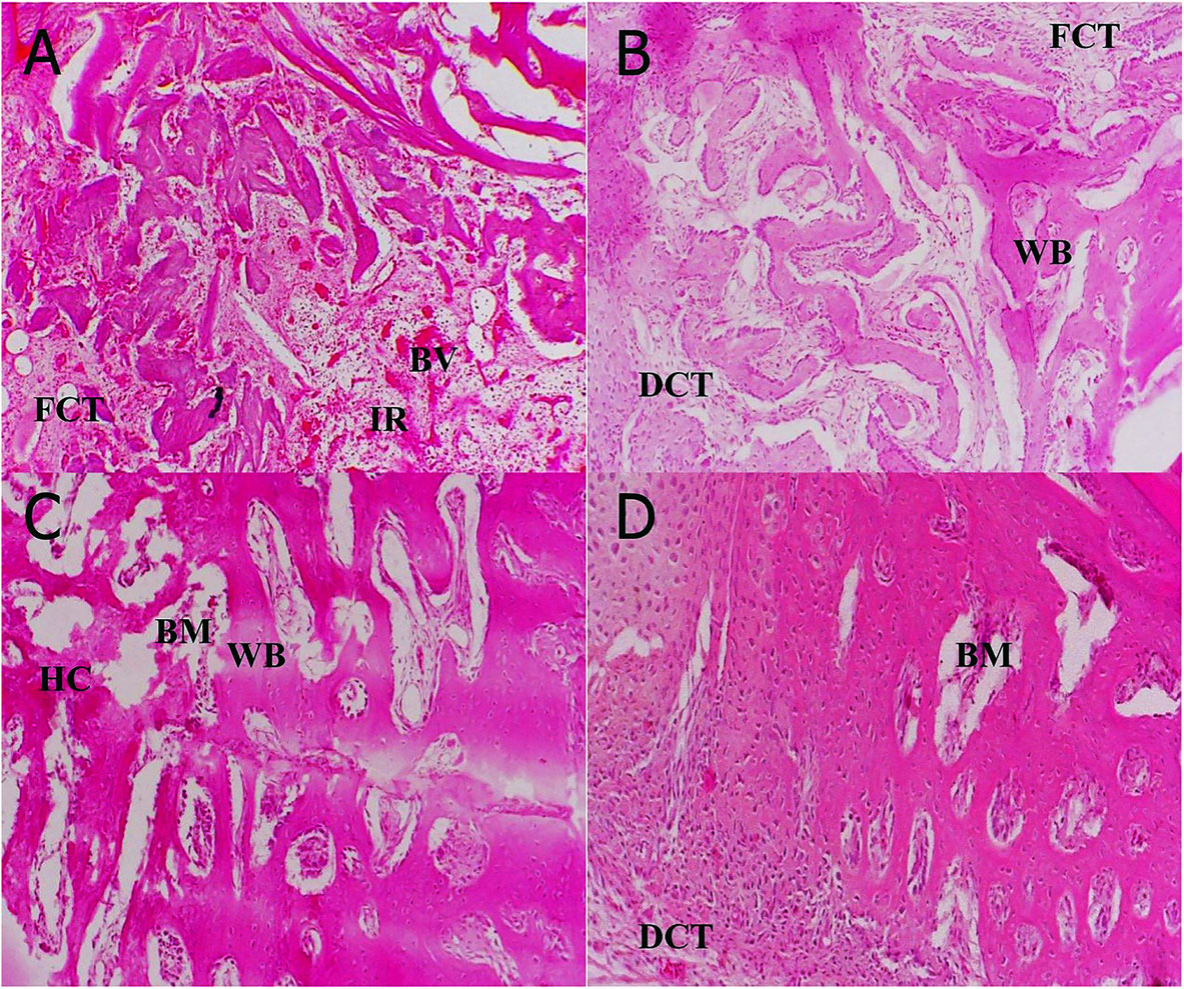
Hematoxylin and eosin (H&E) stained slides of tibial bone tissue for histopathological evaluation (H&E ×100). **A** Control group, **B** PDE 150 group, **C** osteocare group, **D** PDE 300 group. BM: bone marrow, FCT: fibrous connective tissue, DCT: dense connective tissue, IR: inflammatory reaction, HC: hyaline cartilage, BV: blood vessels, WB: woven bone

Histopathological slides of the H&E staining method are indicated in Fig. 4. According to Fig. 4A, in the control group, which did not receive any treatment, a large number of woven bones were observed at the site of repair, and a low reconstruction was observed. Furthermore, large amounts of new vessels were observed, and the amount of hyaline cartilage was lower than the other groups. Weak connective tissue and many single-nucleated inflammatory cells were detected. Figure 4B presents the histopathology of the PDE 150 group. In this group, the formation of primary osteocytes, hyaline cartilage, haversian canals, dense connective tissue was observed at the fracture site. Histopathological slide of the OC group is shown in Fig. 4C. In this treatment group, reconstruction of hyaline cartilage and dense connective tissue was observed simultaneously. Figure 4D indicates a pathological slide regarding the PDE 300 group. New bone formation, very soft connective tissue, formation and regeneration of Haversian canals, and osteocytes’ presence were more notable in the PDE 300 group than that in the other groups.

The results of the correlational analysis of histopathological parameters are summarized in Table 3. The difference between the treatment groups and the control group for all measured parameters were significant except for the inflammatory cells (*P* < 0.05). Treatment with PDE 300 remarkably increased osteoblasts/osteocytes and lamellar bone and diminished woven bone compared to the PDE 150 group (*P* < 0.05). PDE 300 group significantly increased Osteoblasts/Osteocytes in comparison to the OC group (*P* < 0.05).

**Table 3:** Histopathological parameters in different experimental groups of the study

Parameter	Groups			
	Control	PDE 150	PDE 300	OC
Fibroblasts/Fibrocytes	122.5±10.54	51.33±3.06^*^	53.67±1.53^*^	48.33±3.79^*^
Chondroblasts/Chondrocytes	11.33±3.06	59.33±4.61^*^	57.67±14.43*	59.33±3.78^*^
Osteoblasts/Osteocytes	2.33±1.52	42.33±0.57^*^	46.66±1.15^*, **^	42±1^*, ***^
Osteoclast	0.0	1.66±0.57^*^	1.33±0.57^*^	0.66±0.57
Inflammatory cells	13.66±0.57	10±4.58	7.33±5.03	12.33±1.52
Vessels	18±1	12.33±1.52^*^	11±1.73^*^	12.66±2.08^*^
Woven bone	34.67±1.64	28±1.73^*^	20.67±1.53^*, **^	24±5.29^*^
Lamellar bone	6.33±2.3	14.33±2.51^*^	22±2^*, **^	17±6.24^*^

^*^p < 0.05 compared with the control group

^**^p < 0.05 compared with the PDE 150 group

^***^p < 0.05 compared with the PDE 300 group

### Immunohistochemical findings

**Fig. 5:**
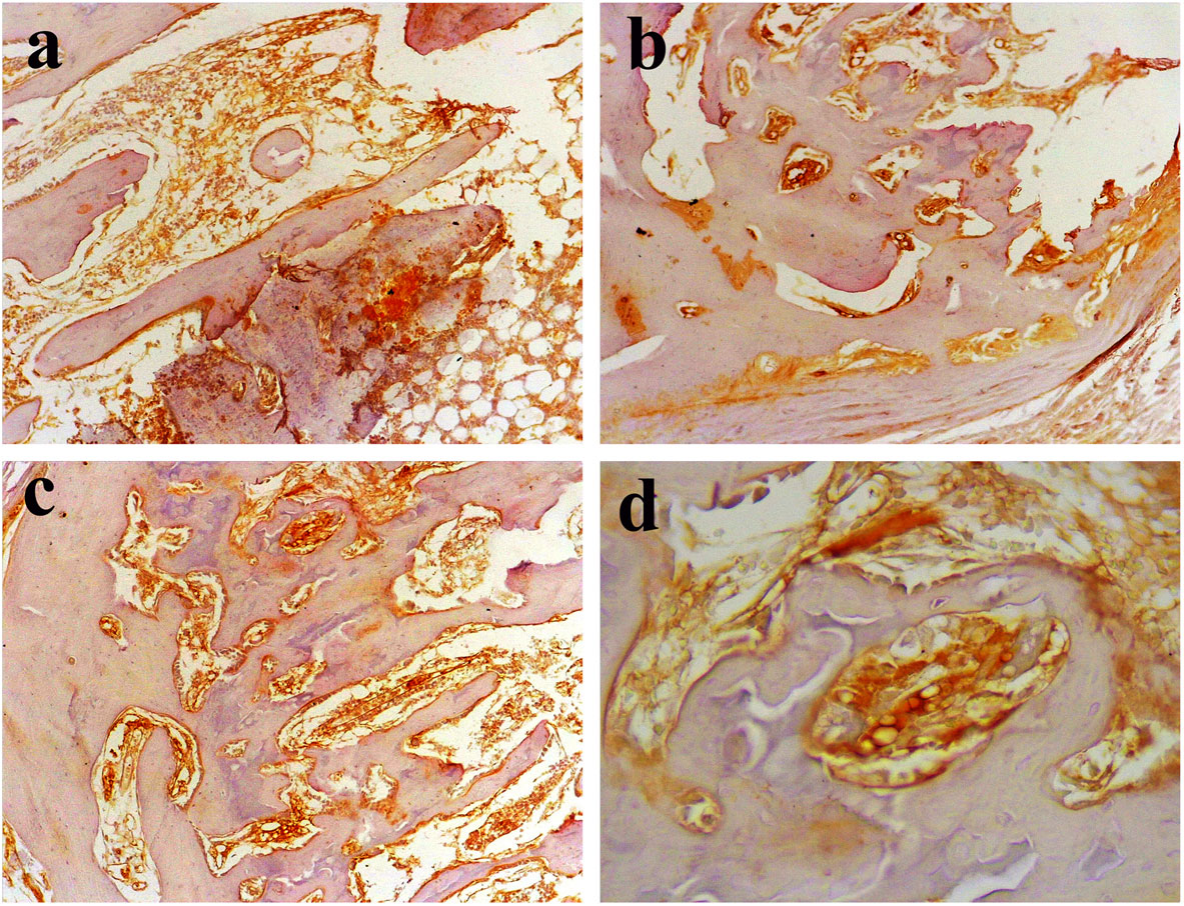
Immunohistochemical stained slides of osteocalcin protein. **a** Control group, **b** PDE 150 group, **c** PDE 300 group, **d** Osteocare group

Figure 5 represents the immunohistochemical stained slides of the osteocalcin in different study groups on day 50 following the euthanizing of animals. Figure 5a shows the control group which did not receive any treatment and osteocalcin marker was highly expressed owing to the activity of the osteoblasts and bone formation on day 50. In other treatment groups, the osteocalcin expression was remarkably lower than that in the control group, showing that the bone formation in the treatment groups was approximately completed on day 50 (Fig. 5b–d).

## Discussion

Bone tissue is made of a matrix of collagen fibers and metabolically active cells. Once the bone is fractured or injured, the healing process is commenced by different mechanisms such as biochemical, biomechanical, cellular, hormonal, and pathological mechanisms [15]. Inflammatory, repair, and remodeling stages are known to be the regenerative pattern of bone healing. These stages are influenced by a variety of systemic and local factors [16]. Parathyroid hormone, vitamin D, and calcitonin play a major role in the systemic regulation of bone remodeling by controlling the levels of serum calcium and alkaline phosphate [16, 17]. Although bone healing is a physiological and spontaneous process, intervention and creating the ideal conditions can be useful for a rapid return to normal function and regeneration [18]. Various methods have been employed in previous studies to speed up the healing process, including electrical stimulation [18], pulsed ultrasound [19], and bone grafts [20]. However, the use of these substances is not economical and not available to the public and requires advanced hospital facilities. Hence, several studies have been performed in bone healing using medicinal plants [6].

Several previous studies have investigated the bone healing process using biochemical markers of the bone formation such as calcium, phosphorus, and alkaline phosphatase [6, 21–24]. High levels of calcium and phosphorus in the blood and extracellular fluids trigger the deposition of calcium phosphate crystals in the osteoid and make it harder [25]. The results of serum calcium in rabbits, on day 10, in the treatment groups revealed a small decrease in serum calcium levels, which coincided with increased blood phosphorus levels, indicating that calcium was being transferred from the blood to the bone. The tibial lesion stimulated the thyroid to secrete calcitonin and deliver more calcium to the site of fracture. Furthermore, following the consumption of *Prunus dulcis* extract, this reduction is more noticeable, which is maybe due to the thyroid reaction to the increment in blood calcium levels [25]. These findings are in agreement with the results of the Florence et al. (2017) study [21]. In another research on treatment by oral supplementation of pomegranate peel extract in a mandibular bone defect in a rabbit, on day 7 post-surgery, the level of calcium decreased [22]. A previous study has reported that the level of blood calcium increases following an increment in the dosage of the Arjuna Terminalia extract, and repair occurs more rapidly in the group with a higher dose of extract. This indicates that blood calcium increment causes more calcium-phosphate crystals to deposit at the fracture site, and repair occurs faster [25]. These results are consistent with those of our study, which suggests that increasing the dosage of the PD extract leads to faster healing in the tibial lesion.

Phosphorus is an essential bone component and is necessary for proper skeletal mineralization. Most of the body’s phosphorus is stored in the bones [26]. Blood phosphorus is initially increased following bone fracture due to necrosis and destruction of bone cells at the fracture site, where the stored phosphorus is released into the blood. Several days after the beginning of the bone healing process, the level of phosphorus in the blood decreases due to the increase in the blood calcium level. Blood phosphorus level is inversely proportional to the calcium [25]. Herein phosphorus serum level in the treatment groups has increased on day 10 after surgery, but in the control group, it was increased on days 30 and 50 post-surgery. It seems that the presence of phosphorus in the *Prunus dulcis* extract accelerates the healing process of bone. Besides, hydroxyapatite crystals in bone are formed by the deposition of phosphorus and calcium in the callus. Therefore, the high amount of phosphorus in the extract speeds up the formation of hydroxyapatite crystals and faster bone healing [21]. In the present study, a faster reduction in the amount of phosphorus was observed in the PDE 300 group compared to the other treatment groups. This can indicate faster hydroxyapatite deposition at the site of bone damage. These findings are in accordance with the previous report by Florence et al. (2017), which showed that P. pellucida ethanolic extract dose-dependently increased mineral deposition [21].

Alkaline phosphatase is an indicator of liver damage or any systemic inflammatory reaction [27]. Alkaline phosphatase is secreted by osteoblasts and plays an important role in bone healing. This enzyme triggers the mineralization of the osteoid by increasing the local concentration of calcium phosphate. Alkaline phosphatase is an indicator for bone formation, and higher serum level of this enzyme shows faster maturation and more activity of osteoblasts [28]. The higher alkaline phosphatase levels in the PDE 300 group and the osteocare group 10 days after surgery indicate greater osteoblast activity, and faster healing.

Shuid et al. (2011) investigated the effect of α-Tocopherol on osteoporotic fracture healing in early stages and concluded that the group treated by α-Tocopherol revealed a significant decrease in the TBARS level and increased the GPx and SOD levels in comparison to the sham group [29]. Effect of tricalcium phosphate/collagen (TCP/Collagen) nanocomposite scaffold on bone healing in a rabbit model was examined by Farahi et al. (2019), and oxidative stress evaluation in the plasma indicated that in the treatment group level of MDA significantly reduced compared to the sham group. SOD and GPx levels significantly increased in the treatment group in comparison to the sham group [27]. In a study conducted by Jia et al. (2006), effects of Almond (*Prunus dulcis*) consumption in the smokers was assessed, and oxidative stress biomarkers including MDA, GPx, and SOD in the plasma were evaluated. In the almond group, the amount of MDA significantly reduced, and levels of GPx and SOD increased, but it was not significant [30]. Another research investigated the protective effects of almond oil on hepatic damage due to carbon tetrachloride. In this research, oxidative stress biomarkers such as SOD, CAT, GPx, and MDA were examined. The authors found that in the treatment group with the higher dosage of almond oil, SOD and CAT significantly increased in comparison to the control group. Moreover, GPx level increased, and MDA level reduced, but the difference was not significant. In agreement with previous studies, in this research, treatment with *Prunus dulcis* extract remarkably reduced MDA level and increased GPx and CAT levels compared to the control group that did not receive any treatment.

In the present study, a comparison of radiographic scores according to the modified Lane and Sandhu method in different study groups on days 10 and 50 showed no significant difference between the treatment groups and the control group. However, on day 30 treatment groups remarkably increased the radiographic score compared to the control group. Oryan et al. (2012) assessed the effects of combined hydroxyapatite and human platelet-rich plasma on radius bone healing in a rabbit. The radiological evaluation based on the modified Lane and Sandhu scoring method revealed that on days 14 and 28 there was no significant difference between the treatment groups and the control group. However, on days 42 and 56, the score in the treatment group was significantly higher than that in the control group [31]. These results are in line with those of our study and suggest that on the 10 first days, treatments do not influence the radiographic scores.

Matos et al. (2008) evaluated the histomorphological parameters of bone healing in a rabbit fibular osteotomy. According to this research, bone healing possesses three histopathological phases. The first stage continues until day 10, and inflammatory cytokines induce intensive cell multiplication in this stage. Two weeks following the fracture, the second phase is commenced with converting the woven bones to the lamellar bone. Eventually, the third stage occurs on day 21, and cartilage and woven bone can be observed around the callus as an evidence of this stage [32]. Matos et al. (2008), in this research, evaluated the percentage of woven and lamellar bone in the first, second, and fourth week during fracture healing and concluded that the percentage of woven bone in the first week was significantly higher than the lamellar bone and in the fourth week, the percentage of lamellar bone notably increased compared to the woven bone [32]. In accordance with previous results, in the present research on day 50, a significant increase in the numbers of chondroblasts/chondrocytes, osteoblasts/osteocytes, and lamellar bone was observed in the treatment groups in comparison to the control group. Among treatment groups, the PDE 300 group significantly increased the number of osteoblasts/osteocytes compared to the OC group.

Wang et al. (2009) in a research examined the effects of VEGF on osteogenesis following shockwave-promoted fracture healing in rabbits and concluded that osteocalcin expression after 56 days was significantly higher in the control group than that in the treated group [33]. In accordance with the previous results, the present study demonstrated that the treatment groups significantly reduced the osteocalcin expression compared to the control group on day 50. This result may be explained by the fact that in the control group on day 50 the healing process is in progress and osteoblasts are active but in the treatment groups particularly in the PDE 300 and the OC group, bone regeneration is approximately complete on day 50.

## Conclusion

The main goal of the current study was to determine the treatment effects of aqueous extract of *Prunus dulcis* on bone healing in the rabbit. The beneficial effects of the extract on blood biochemical parameters, radiological assessments, histological, and IHC evaluations may be owing to the mineral composition of the extract and the antioxidant effects, as suggested by the assessment of oxidative stress biomarkers. The major limitation of this study is that the molecular detection of bone healing biomarkers was not assessed.

## Data Availability

All data generated or analyzed during this study are included in this published article.

## Abbreviations

MDA: Malondialdehyde
GPx: Glutathione peroxidase
CAT: Catalase
IHC: Immunohistochemistry
PDE: *Prunus dulcis* extract
OC: Osteocare
